# A mechanobiological hypothesis on bone cement-induced progression of bone metastases

**DOI:** 10.3389/fbioe.2026.1766264

**Published:** 2026-05-12

**Authors:** Qiyu Sun, Shuai Li, Zhiqian Sun, Xiaolin Pan, Juan Shen, Yanbo Hu, Dandan Zhao, Lulu Du, Zhen Hao, Xiaowen Ma, Min Li

**Affiliations:** 1 The Postgraduate Training Base of Jinzhou Medical University (The 960th Hospital of PLA), Jinan, China; 2 The 960th Hospital of PLA, Jinan, China; 3 Shandong Second Medical University, Weifang, China; 4 Department of Cadre Diagnosis and Treatment, Eighth Medical Center, Chinese PLA General Hospital, Beijing, China

**Keywords:** bone metastases, hypothesis, mechanobiology, mechanotransduction pathways, PMMA cement, tumor progression

## Abstract

We propose the hypothesis that long-term alterations in the local biomechanical environment (a significant increase in stiffness and changes in stress distribution) induced by the implantation of bone cement for treating bone metastases may, by activating mechanotransduction pathways in tumor cells, potentially promote their proliferation, invasion, and therapy resistance, thereby affecting long-term disease progression. This effect is particularly noteworthy in specific patient populations with extended survival, low tumor burden, and radiosensitivity. For such patients without immediate structural instability risks, prioritizing non-invasive treatments like stereotactic radiotherapy can achieve effective symptom relief while avoiding the potential adverse biological effects associated with altering the bone’s natural mechanical properties, which may lead to better long-term tumor control. This hypothesis does not challenge the critical role of bone cement in managing acute or impending pathological fractures. Instead, it advocates for a reassessment of its status as the “default” treatment option in certain specific patient populations, aiming to promote a more comprehensive balance between short-term symptom relief and long-term disease modulation in clinical decision-making.

## 1 Introduction

Bone metastasis represents a major clinical challenge in the field of oncology, affecting approximately 70% of patients with advanced breast and prostate cancer (Li B. et al., 2022), with an incidence rate reaching 30%–40% in patients with other solid tumors (Khanmohammadi et al., 2024). The traditional treatment focus for metastatic bone disease lies in the palliative management of symptoms, particularly pain relief and the prevention of skeletal-related events (SREs), such as pathological fractures, spinal cord compression, and hypercalcemia (Yang et al., 2022). Among the various existing treatment modalities, polymethylmethacrylate (PMMA) bone cement augmentation techniques—minimally invasive image-guided procedures in which bone cement is injected into weakened metastatic bone to improve mechanical stability and relieve pain—including vertebroplasty, kyphoplasty, and cementoplasty, are widely recognized as effective interventions (Chao et al., 2023). The clinical success of bone cement in the treatment of painful bone metastases has been well established. Reported pain relief rates often exceed 80%–90%, and the procedure can provide immediate mechanical stabilization (Chao et al., 2023; Hovenier et al., 2025). Current clinical guidelines primarily recommend bone cement augmentation for selected patients with painful metastatic vertebral lesions associated with mechanical instability, vertebral compression fracture, or high risk of pathological fracture, particularly in the context of vertebroplasty or kyphoplasty (Barcena et al., 2025; Lyu et al., 2025). This procedure offers several advantages: it is minimally invasive, provides rapid symptom relief, allows for treatment of multiple lesions in a single session, and has broad applicability for patients unsuitable for major surgery (Zhang et al., 2024; Gumusay et al., 2022). Consequently, the use of bone cement has expanded significantly over the past 2 decades, with thousands of such procedures performed globally each year. However, the widespread adoption of bone cement augmentation has been largely driven by its significant short-term benefits (symptom control and mechanical support). Most clinical studies focus primarily on outcomes within 6–12 months post-intervention. Given that the median survival of patients with bone metastases has historically been limited, often measured in months rather than years, this emphasis on immediate and short-term gains is understandable. Within this context, the primary therapeutic goal is to maximize quality of life during the remaining time, making rapid pain relief and functional improvement paramount considerations. Bone cement augmentation provides immediate structural stabilization, but it also introduces a persistent alteration in the local mechanical environment. As survival improves in selected patients with bone metastases, the long-term biological consequences of this altered biomechanical milieu deserve closer scrutiny.

### 1.1 The central hypothesis

We hypothesize that, although bone cement augmentation remains an important and often appropriate treatment for bone metastases associated with mechanical instability, impending pathological fracture, or established fracture, its use as the default treatment for all symptomatic bone metastases warrants reconsideration. In selected patients without immediate structural instability—particularly those with limited disease burden, longer expected survival, and radiosensitive tumors—the persistent biomechanical alterations induced by bone cement may have unintended mechanobiological consequences, and radiotherapy-based approaches may therefore be preferable as an initial local treatment strategy.

## 2 Biomechanical alterations induced by bone cement and their potential biological implications

### 2.1 Magnitude and nature of mechanical environment changes

**FIGURE 1:**
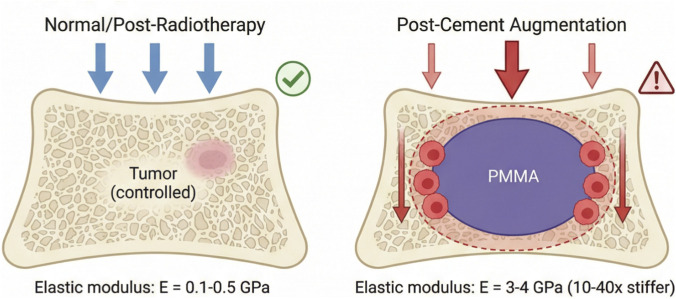
Schematic Illustration of Biomechanical Alterations Induced by Bone Cement and Their Potential Biological Significance. Normal/Post-Radiotherapy Vertebrae: Elastic modulus E = 0.1–0.5 GPa. Cement-Augmented Vertebrae: Elastic modulus E = 3–4 GPa (showing a 10- to 40-fold increase).

The insertion of PMMA bone cement fundamentally transforms the mechanical landscape of the treated bone region (Wang et al., 2021) (Figure 1). This transformation operates across multiple spatial scales and involves several distinct but interrelated mechanical phenomena. Figure 1 summarizes the magnitude of the stiffness mismatch created by bone cement augmentation and highlights the resulting changes in load transfer and local mechanical environment. It serves as a conceptual introduction to the biomechanical changes discussed in Section 1.

### 2.2 Stiffness mismatch and its consequences

The most obvious mechanical change is the dramatic difference in material stiffness. While normal cancellous bone has an elastic modulus ranging from 0.1 to 0.5 GPa and cortical bone ranges from 10 to 20 GPa, PMMA cement exhibits an elastic modulus of approximately 3–4 GPa (Kim et al., 2025). In metastatic bone disease, where osteolytic lesions have already compromised structural integrity, the local bone stiffness may be even lower than normal values. The introduction of cement thus creates a rigid inclusion within a relatively compliant matrix—or in the case of vertebral augmentation, a stiff core within a weakened vertebral body. This stiffness mismatch has several mechanical consequences:

Within the cemented region, the rigid cement bears a disproportionate share of applied loads, effectively shielding the surrounding bone from normal physiological stresses. This stress deprivation is well-known to trigger bone resorption through mechanobiological feedback mechanisms (Wolff’s Law), potentially accelerating bone loss in already compromised regions (Wang et al., 2025).

At the cement-bone interface and in regions immediately adjacent to the cement mass, stress concentrations arise due to the abrupt transition in material properties. Finite element analyses have consistently demonstrated peak stresses at these interface zones, which may be several-fold higher than stresses in normal bone under equivalent loading conditions (van Duren et al., 2025). These zones of elevated stress represent regions of altered mechanical stimulation that may influence cellular behavior (Chougule et al., 2024). The cement mass changes the pathways through which loads are transmitted through the skeleton (Chen X. et al., 2025). In vertebral augmentation, for example, the rigid cement core alters the complex load-sharing between cortical shell and trabecular centrum. In long bone cementoplasty, load distribution patterns across articular surfaces and through the diaphysis are modified (Lyu et al., 2025; Fereydoonpour et al., 2026). These changes affect not only the treated level but also adjacent structures, as evidenced by the increased risk of adjacent vertebral fractures following vertebroplasty.

### 2.3 Dynamic vs. static mechanical environment

Beyond quasi-static stiffness changes, bone cement alters the dynamic mechanical environment. Normal bone exhibits viscoelastic behavior, with energy dissipation and time-dependent mechanical response. Trabecular bone, in particular, undergoes continuous microdamage and repair under cyclic loading (Smotrova et al., 2024). The cement, being a synthetic polymer with different viscoelastic properties, changes the dynamic response of the composite structure. During normal daily activities—walking, sitting, standing, postural changes—bones experience complex, time-varying loads. The cemented region responds differently to these dynamic loads compared to normal or even tumor-affected bone (Zhao et al., 2023). The frequency content of mechanical stimulation, the rate of stress application, and the cyclic nature of loading all influence cellular mechanotransduction. By altering these dynamic characteristics, cement may change the nature of mechanical signals perceived by cells in and around the treated region.

### 2.4 Microenvironmental changes beyond pure mechanics

While our focus is on mechanical factors, it is important to acknowledge that cement insertion also induces several non-mechanical changes that may interact with or compound mechanical effects:

The exothermic polymerization of PMMA generates temperatures that can reach 70 °C–100 °C at the cement-bone interface (Kim et al., 2025). While this thermal pulse is transient (lasting minutes), it causes local tissue damage, protein denaturation, and cell death within a zone extending several millimeters from the cement. Paradoxically, this thermal effect is often cited as potentially beneficial for local tumor control (thermal ablation), but it also damages normal tissue including blood vessels, bone cells, and potentially immune cells. The long-term consequences of this acute thermal insult on the surviving cell populations and tissue repair processes remain poorly characterized.

Chemical microenvironment: The monomer used in PMMA preparation (methyl methacrylate) has known cytotoxic effects, and residual monomer may persist in tissues for days to weeks (Seesala et al., 2024). Additionally, cement degradation products, though minimal for PMMA, may influence the local chemical environment over time (Wang et al., 2021). The interface between cement and bone becomes a site of chronic foreign body reaction, with macrophage accumulation and low-grade inflammation that persists indefinitely (Panez-Toro et al., 2023).

Cement injection often compromises local microvasculature through direct mechanical disruption, thermal damage, and mass effect. While this vascular injury may help control tumor (which requires angiogenesis), it also impairs delivery of systemic therapies, immune cell trafficking, and normal bone remodeling.

The cement mass physically occupies space previously filled with bone marrow, tumor, and vascular tissue. This creates a barrier to cellular migration and fluid transport, potentially compartmentalizing the treated region and altering drug distribution and immune surveillance.

These multiple factors—mechanical, thermal, chemical, and vascular—operate simultaneously and may interact in complex ways. However, while thermal and chemical effects are transient, and vascular changes stabilize within weeks to months, the mechanical alterations persist indefinitely as long as the cement remains in place. It is this chronic, persistent change in mechanical environment that forms the basis of our hypothesis regarding long-term tumor behavior.

## 3 Mechanobiology of cancer: how physical forces influence tumor behavior

**FIGURE 2:**
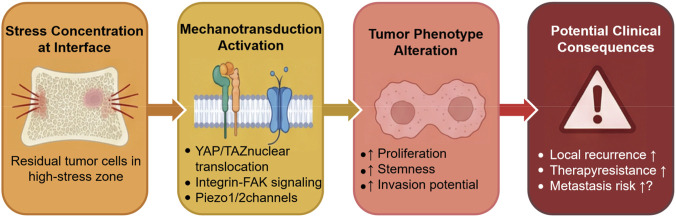
Vertebral Mechanotransduction Pathway Schematic. Stress concentration within the vertebra activates mechanotransduction pathways (e.g., YAP/TAZ transcription factors), leading to altered tumor phenotypes that impact potential clinical outcomes of bone metastases.

The past 2 decades have witnessed a revolution in our understanding of how physical forces regulate cell behavior and tissue organization. It is now well-established that cells do not simply respond to biochemical signals; they actively sense and respond to mechanical cues from their environment (Cao et al., 2024) (Figure 2). This field—mechanobiology—has profound implications for understanding cancer.

To provide a conceptual bridge between the altered mechanical environment created by bone cement and its possible biological consequences, we summarize the proposed mechanotransduction framework in Figure 2. The figure illustrates how local stress concentration and stiffness changes may activate intracellular signaling pathways, including integrin-associated signaling and YAP/TAZ-related transcriptional responses, which could ultimately influence tumor proliferation, invasion, and treatment resistance. These candidate mechanisms are discussed in greater detail in the following sections.

### 3.1 Mechanotransduction: From force to biochemical signal

**FIGURE 3:**
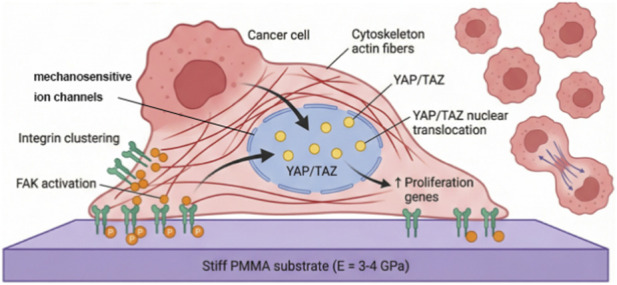
Cellular mechanotransduction pathways potentially activated by cement-induced stress concentration in bone metastases. Mechanical stimuli may be sensed through integrin-based adhesions, mechanosensitive ion channels, cytoskeletal tension, and nuclear force transmission, leading to downstream signaling changes that influence tumor behavior.

Cells convert mechanical stimuli into biochemical signals through a process called mechanotransduction (Figure 3). Figure 3 summarizes the principal candidate mechanotransduction routes discussed in this section, including integrin-associated adhesions, mechanosensitive ion channels, cytoskeletal force transmission, and nuclear mechanotransduction. Cells convert mechanical stimuli into biochemical signals through several interconnected mechanotransduction mechanisms. The principal candidate pathways relevant to our hypothesis are summarized below.

Integrin-based adhesions are among the best-characterized mechanosensing structures. Integrins physically connect the extracellular matrix to the intracellular cytoskeleton. When mechanical forces are applied to the matrix, integrins cluster and recruit signaling proteins such as focal adhesion kinase (FAK), Src, and adaptor molecules, thereby activating downstream pathways including PI3K/AKT, MAPK/ERK, and RhoA/ROCK. These signaling cascades regulate cell proliferation, survival, and motility. (Chen Z. et al., 2023; Diaz-Palacios et al., 2025).

The Hippo-YAP/TAZ pathway represents another major mechanotransduction route in cancer biology. YAP and TAZ are mechanosensitive transcriptional regulators that translocate to the nucleus in response to increased matrix stiffness, mechanical stretch, or altered cell-cell contact. Once activated, they promote the expression of genes associated with proliferation, survival, and stem-like phenotypes, and their activity has been linked to cancer progression and metastasis. (Li H. et al., 2022; Piccolo et al., 2023).

Mechanosensitive ion channels and membrane mechanics provide an additional layer of force sensing. Channels such as Piezo1/2 and members of the TRP family can open in response to membrane tension or deformation, leading to calcium influx and downstream signaling activation. Because calcium signaling influences many aspects of cell behavior, this pathway may complement integrin- and YAP/TAZ-mediated responses in mechanically altered tumor microenvironments. (Karkempetzaki and Ravid, 2024; Harraz et al., 2022).

Nuclear mechanotransduction further extends this process by transmitting force directly to the nucleus through the LINC complex and associated cytoskeletal structures. Mechanical deformation of the nucleus can alter chromatin organization, transcription factor accessibility, and epigenetic regulation, thereby influencing gene expression in a more direct and sustained manne (Amiad Pavlo et al., 2023). Nuclear deformation under mechanical stress can affect transcription factor access to DNA and epigenetic modifications (Kalukula et al., 2022).

### 3.2 Matrix stiffness and cancer progression: Experimental evidence

The relationship between tissue stiffness and cancer has been most extensively studied in breast cancer, where increased mammographic density (a surrogate for tissue stiffness) is an independent risk factor for cancer development (Lundberg et al., 2024). Experimental studies have demonstrated causative relationships: When cultured on substrates of varying stiffness, many cancer cell types exhibit stiffness-dependent behaviors. On stiff substrates (mimicking tumor tissue, typically >5 kPa), cells display increased proliferation, reduced apoptosis, enhanced migration, and more invasive morphology compared to soft substrates (mimicking normal tissue, typically 0.5–2 kPa) (Rubí-Sans et al., 2025). These effects are mediated by the mechanotransduction pathways described above, particularly integrin-FAK and YAP/TAZ signaling (Chuang and Ito, 2021). In mouse models, increasing matrix stiffness through collagen crosslinking or fibrosis promotes tumor growth and metastasis (Peng et al., 2025). Conversely, pharmacological reduction of matrix stiffness (using LOXL inhibitors to prevent collagen crosslinking) can inhibit tumor progression (Ferreira et al., 2021). These studies establish that matrix stiffness is not merely a consequence of tumor progression but an active driver of malignant behavior.

In human cancers, tissue stiffness measured by elastography correlates with aggressive features and poor prognosis in breast, liver, and pancreatic cancers (Liu et al., 2021). High-stiffness regions within tumors often harbor more aggressive cell populations with stem-like properties (Safaei et al., 2023).

### 3.3 Mechanical forces in metastasis

Mechanical factors influence multiple steps of the metastatic cascade:

Local invasion: Increased ECM stiffness promotes invasive protrusions and matrix degradation. Stiff matrices enhance expression of matrix metalloproteinases (MMPs) and promote epithelial-mesenchymal transition (EMT), facilitating local invasion (Arai et al., 2021).

Intravasation and circulation: During transit through the bloodstream, circulating tumor cells (CTCs) experience extreme mechanical stresses from fluid shear forces and physical confinement in capillaries (Xin et al., 2024). Cells that survive these mechanical challenges tend to have altered mechanical properties (increased deformability) and activated survival signaling (Chen et al., 2022). Mechanical stress during circulation can induce epigenetic changes that enhance metastatic potential.

Extravasation and colonization: After arrest within the microvasculature of a distant organ, circulating tumor cells must adhere to the endothelium, deform, and traverse the endothelial barrier and basement membrane in order to extravasate into the surrounding tissue. This step is also influenced by mechanical factors, including vascular confinement, shear stress history, and the deformability of tumor cells. Once tumor cells enter the metastatic site, the mechanical properties of the local tissue then influence colonization efficiency. Bone, with its unique mechanical environment and constant remodeling, provides a particularly complex mechanical niche for metastatic cells (Kumar et al., 2024).

### 3.4 The bone microenvironment: a special case

Bone is perhaps the most mechanically active tissue in the body, constantly remodeling in response to mechanical loads. The bone marrow microenvironment combines structural cells (osteoblasts, osteocytes, osteoclasts), hematopoietic cells, adipocytes, and stromal cells in a three-dimensional mineralized matrix subject to complex mechanical stresses (Chen et al., 2021). Within this environment, osteocytes serve as key mechanosensors, detecting mechanical strain and coordinating bone remodeling through factors such as RANKL, OPG, and sclerostin (Liu et al., 2025; Yan et al., 2023). This mechanical regulation is fundamental to skeletal homeostasis. Tumor cells in bone engage in a well-described “vicious cycle” with bone cells. Tumor cells secrete factors (PTHrP, IL-6, RANKL) that stimulate osteoclasts, causing bone resorption. This releases growth factors from the bone matrix (TGF-β, IGFs, BMPs) that further stimulate tumor cells (Li et al., 2025; Yang et al., 2024). Mechanical forces modulate multiple components of this cycle—mechanical loading affects both osteoblast and osteoclast activity, and can influence tumor cell behavior directly (Siddiqui et al., 2022). The bone marrow “niche” that supports hematopoietic stem cells and metastatic tumor cells is mechanically regulated. Substrate stiffness influences stem cell fate decisions and self-renewal capacity. There is growing evidence that mechanical signals help define the pre-metastatic and metastatic niches in bone (Kumar et al., 2024).

## 4 Potential mechanisms linking cement-induced mechanical changes to tumor progression

Bringing together the biomechanical alterations caused by cement and the mechanobiological principles outlined above, we can propose several plausible mechanisms by which cement insertion might influence tumor behavior.

### 4.1 Hypothesis 1: direct mechanotransduction in residual tumor cells

Cement augmentation is rarely if ever completely ablative—viable tumor cells typically remain at the periphery of the cemented region and in adjacent bone. These residual cells now exist in a dramatically altered mechanical environment, with substantially increased local stiffness and altered stress patterns. Based on the mechanobiology literature, we would predict that residual tumor cells exposed to this stiffer environment might exhibit:Enhanced YAP/TAZ nuclear localization and activity, promoting proliferation and stem-like properties. Increased integrin-FAK signaling, enhancing survival and therapy resistance. Altered sensitivity to systemic therapies due to changes in drug penetration and cell state. Enhanced invasive potential due to EMT induction. These effects would be most pronounced in cells at the cement-bone interface, where stress concentrations are highest, and might manifest as preferential tumor recurrence in peri-cement regions.

### 4.2 Hypothesis 2: mechanical modulation of the bone microenvironment

Even if cement does not directly contact tumor cells, it alters the mechanical state of surrounding bone tissue, which may indirectly affect tumor behavior through changes in bone cell activity:

Stress shielding effects: Reduced mechanical loading in cement-adjacent bone may decrease osteoblast activity and increase osteoclast activity (disuse osteoporosis), potentially tipping the balance of the vicious cycle toward more bone resorption and tumor-promoting factor release.

Stress concentration effects: Conversely, regions of elevated stress may experience increased bone turnover, creating a more dynamic microenvironment that could either suppress or promote tumor growth depending on the specific context.

Disruption of mechanobiological homeostasis: The normal balance between mechanical loading, bone remodeling, and growth factor release is disrupted, potentially creating a microenvironment that favors tumor persistence or progression.

### 4.3 Hypothesis 3: compromised immune surveillance

Emerging evidence suggests that mechanical forces influence immune cell function and tumor immunology:Matrix stiffness affects T cell trafficking, activation, and effector function (Hyun et al., 2023; Zhang et al., 2025). Mechanical stress can induce immunosuppressive changes in the tumor microenvironment (Juthi et al., 2025; Lei et al., 2021). The chronic inflammation associated with cement (foreign body reaction) may create an immunosuppressive milieu. Vascular disruption impairs immune cell recruitment to tumor regions. These factors could reduce the effectiveness of both endogenous anti-tumor immunity and immunotherapy approaches.

### 4.4 Hypothesis 4: altered response to systemic therapies

The mechanical environment influences cancer cell phenotype and drug sensitivity:Stiff matrices can induce a more stem-like, therapy-resistant phenotype. Changes in vascular perfusion affect drug delivery. Mechanical stress can activate survival pathways that antagonize therapy-induced apoptosis. The physical barrier of cement may create pharmacokinetic sanctuaries. Patients treated with cement might therefore show altered responses to subsequent systemic therapies compared to patients treated with radiotherapy alone. These hypotheses are not mutually exclusive and may operate simultaneously. Moreover, the relative importance of each mechanism likely depends on specific factors including tumor type, extent of disease, cement volume and location, and patient-specific biomechanics. What they share is a common foundation: the recognition that mechanical forces matter in cancer biology, and that cement-induced mechanical changes may have biological consequences beyond immediate structural stabilization.

## 5 Reinterpreting existing clinical evidence through a mechanobiological lens

The clinical literature on bone cement for metastatic disease is extensive, with hundreds of published studies reporting outcomes (Chao et al., 2023; Wang et al., 2022). However, these studies were designed primarily to evaluate short-term efficacy (pain relief, functional improvement) and procedural safety (cement leakage, complications) (Liu et al., 2024; Brokke et al., 2025). The question of whether cement-induced mechanical changes influence long-term tumor behavior has rarely been explicitly addressed. Nevertheless, a critical reexamination of existing evidence through the lens of mechanobiology reveals several intriguing observations and identifies important knowledge gaps.

### 5.1 Limitations of current clinical evidence

#### 5.1.1 Short follow-up periods

The majority of studies evaluating cement augmentation for bone metastases report outcomes over 3–12 months (Chao et al., 2023). While this timeframe is appropriate for assessing pain relief and quality of life—and may encompass the entire remaining lifespan for many patients with advanced disease—it is insufficient to detect subtle effects on tumor biology that might manifest over longer periods. Consider the mechanobiological mechanisms we have proposed: altered mechanotransduction signaling, changes in bone remodeling, modulation of dormancy, and effects on therapy resistance. These processes likely operate over months to years rather than weeks. If cement-induced mechanical changes increase local tumor growth rates by, for example, 20%–30%, this might not become radiographically apparent until 12–18 months post-procedure—beyond the follow-up period of most studies. Furthermore, many patients receive systemic therapies that evolve over time, making it difficult to attribute changes in disease trajectory to any single local intervention. The signal of a mechanobiological effect could easily be obscured by the noise of changing systemic treatments, disease heterogeneity, and competing risks in this population.

#### 5.1.2 Endpoints and outcome measures

Local tumor progression is seldom used as a primary endpoint in studies of cement augmentation. Instead, most studies focus primarily on short-term clinical outcomes, such as pain scores (e.g., VAS and NRS), quality-of-life measures (e.g., EORTC QLQ and EQ-5D), functional status (e.g., Karnofsky Performance Status and ambulatory capacity), and procedure-related complications, including cement leakage, fracture, and infection. When local disease control is evaluated, it is usually treated as a secondary endpoint and is often defined inconsistently. Some studies report “local recurrence” without clearly specified radiographic criteria, whereas others rely on subjective radiological assessment without standardized measurement protocols. Only a small number of studies use quantitative volumetric analysis or advanced imaging modalities such as PET-CT, which may be more sensitive for detecting subtle metabolic changes before overt structural progression becomes apparent. In addition, in patients with widespread metastatic disease, it can be difficult to determine whether progression at a cemented site reflects true local recurrence or the development of a new metastatic focus. Retrospectively, it is even more challenging to distinguish between disease that was insufficiently controlled at baseline and disease whose subsequent behavior may have been altered by changes in the local microenvironment.

#### 5.1.3 Selection bias and confounding

Clinical studies of cement augmentation are subject to several forms of selection bias that complicate interpretation. Indication bias is particularly important, as patients undergoing cement augmentation often have more severe symptoms, greater structural compromise, or prior treatment failure, all of which are independently associated with worse outcomes. In contrast, patients managed conservatively or with radiotherapy alone may have less aggressive disease or better performance status, making direct comparisons inherently problematic. Immortal time bias may also arise because patients must survive long enough and remain fit enough to receive the procedure, which can spuriously favor the cement-treated group in survival analyses. In addition, treatment era effects are difficult to exclude, since the increasing use of cement augmentation has coincided with major advances in systemic therapy. As a result, comparisons between historical non-cement cohorts and more contemporary cement-treated cohorts may reflect broader changes in oncologic care rather than the isolated effect of cement augmentation itself.

#### 5.1.4 Lack of mechanistic investigation

Perhaps most importantly, clinical studies have not been designed to test mechanobiological hypotheses. We lack data on:Mechanotransduction pathway activation in peri-cement tissues. Bone remodeling markers before and after cement insertion. Detailed imaging characterization of the cement-bone interface over time. Correlation between mechanical parameters (cement volume, location, interface stress) and biological outcomes. Circulating tumor cell dynamics or molecular markers of mechanical stress responses. Without such mechanistic data, even well-designed clinical studies cannot definitively address whether mechanical factors influence tumor behavior.

### 5.2 Short-term efficacy does not resolve long-term oncological uncertainty

Current clinical evidence supports the short-term palliative benefit of bone cement augmentation, particularly in terms of pain relief and mechanical stabilization (Xu et al., 2024; Jahani et al., 2025).

### 5.3 Clinical equipoise and the rationale for reconsideration

The reinterpretation above is not meant to indict current practice or suggest that cement is harmful. Rather, it highlights that: Our clinical evidence base is optimized for assessing short-term palliative efficacy, not long-term oncological outcomes. Several observations in the existing literature are at least consistent with mechanobiological effects, even if not conclusive. For specific patient populations (long expected survival, limited disease, radiation-sensitive tumors), clinical equipoise exists regarding the optimal local treatment approach. A mechanobiologically informed treatment selection strategy may represent a useful area for future investigation in these selected patients. This framework supports prospective study of whether radiotherapy-based sequencing could offer advantages in appropriately selected clinically stable patients, while recognizing that current evidence does not justify a change in established practice.

## 6 Levels of evidence supporting the present hypothesis

To avoid overinterpretation, it is important to distinguish between findings that are directly supported by existing evidence and those that remain inferential or hypothetical. The present manuscript brings together biomechanical reasoning, mechanobiology, and clinical observations, but the strength of evidence is not uniform across these domains. First, there is substantial experimental evidence that mechanical properties of the cellular microenvironment, including matrix stiffness, stress, and strain, can regulate tumor cell behavior through mechanotransduction pathways (Chai et al., 2023; Chen H. et al., 2025). In particular, the relationships between extracellular stiffness and signaling pathways such as integrin-associated signaling and Hippo-YAP/TAZ are well supported in the broader mechanobiology literature (Zeng et al., 2022). Likewise, the ability of bone cells and the bone microenvironment to respond to mechanical stimuli is well established. These findings provide a strong biological basis for considering whether persistent cement-induced mechanical changes might have downstream biological consequences. Second, there is also established clinical evidence that bone cement augmentation provides effective short-term pain relief and mechanical stabilization in selected patients with metastatic bone disease, especially in the setting of structural compromise, instability, or fracture risk (Sun et al., 2025; Primayudha et al., 2025). Similarly, radiotherapy-based approaches are well recognized as important treatment options for symptom control and local disease management in appropriate clinical scenarios (Lee et al., 2025; Hoeltgen et al., 2023). However, the existing clinical literature has largely focused on short-term palliative outcomes, functional improvement, and procedural safety, rather than on long-term mechanobiological or oncological consequences of altered local stiffness after cement augmentation (Al Raaisi et al., 2022). Third, the proposition that bone cement-induced biomechanical alterations may directly promote tumor progression, invasiveness, or treatment resistance should be regarded as a hypothetical extrapolation rather than an established clinical fact. This component of the argument is not currently supported by direct prospective clinical evidence. Instead, it is inferred from the convergence of three observations: (1) bone cement can create persistent changes in local stiffness and stress distribution; (2) mechanical cues can influence tumor and stromal cell behavior in experimental systems; and (3) current clinical studies have not been designed to detect these possible long-term biological effects. Accordingly, the present manuscript should be interpreted as proposing a biologically plausible but as yet unproven clinical hypothesis. From this perspective, the main purpose of this article is not to claim that cement augmentation has been shown to worsen oncological outcomes, but rather to clarify why this possibility deserves further investigation. Future work will be required to determine whether the mechanobiological effects proposed here are clinically meaningful, which patient subgroups may be most relevant, and whether alternative treatment sequencing strategies could improve long-term outcomes in selected clinically stable patients.

A key interpretive limitation of the present hypothesis is that much of the mechanobiology literature linking matrix stiffness to malignant behavior has been derived from soft-tissue tumor models, including breast cancer and other epithelial malignancies, rather than from metastatic tumors growing within bone. Although these studies provide important conceptual support for the idea that mechanical cues can regulate tumor cell behavior, bone represents a highly distinctive mechanobiological environment that cannot be reduced to a simple stiffness model. Unlike soft tissues, bone contains a mineralized extracellular matrix, a dense and spatially heterogeneous architecture, and a resident cellular network of osteocytes, osteoblasts, and osteoclasts that continuously sense and respond to mechanical loading. In addition, bone metastases develop within a dynamic niche characterized by ongoing remodeling, release of matrix-stored growth factors, and reciprocal interactions between tumor cells and skeletal cells. These features mean that the biological consequences of altered stiffness in bone may not mirror those observed in soft-tissue experimental systems. Accordingly, the present manuscript does not assume a direct one-to-one transfer of soft-tissue mechanobiology findings to metastatic bone disease. Rather, it uses these findings as a conceptual starting point to formulate a biologically plausible question: whether persistent cement-induced changes in the local mechanical environment of bone could influence tumor behavior in ways that have not yet been adequately studied. This extrapolation should therefore be interpreted cautiously and requires dedicated validation in bone-specific experimental models and prospective clinical investigations.

## 7 Alternative treatment approaches: radiotherapy as a principal alternative

If cement-induced mechanical alterations potentially influence tumor biology, what are the alternatives for patients who do not have immediate structural failure requiring urgent mechanical stabilization? This section examines radiation-based approaches and their advantages from a mechanobiological perspective.

### 7.1 Radiotherapy: preserving mechanical homeostasis while achieving tumor control

External beam radiotherapy or brachytherapy has been a cornerstone of bone metastasis management for decades. Unlike cement, radiation does not fundamentally alter the mechanical environment of bone; rather, it works within the existing biomechanical framework to achieve local tumor control.

#### 7.1.1 Mechanisms of action and biological effects

Radiotherapy achieves tumor control through: Direct DNA damage causing cell death (apoptosis, mitotic catastrophe). Vascular damage reducing tumor perfusion. Immune activation through release of tumor antigens and danger signals. Inhibition of tumor-associated osteoclast activity, reducing bone destruction. Importantly, radiation also promotes bone healing in osteolytic lesions: Reduction of tumor burden allows osteoblastic repair. Decrease in tumor-secreted factors that drive osteoclastogenesis. Restoration of more normal bone remodeling balance. Potential for actual remineralization and structural recovery over months. From a mechanical perspective, successful radiotherapy can gradually restore bone structure and mechanical properties rather than replacing bone with a synthetic material of different mechanical characteristics. This represents a fundamentally different biomechanical trajectory.

#### 7.1.2 Evidence for efficacy in pain control

Multiple randomized controlled trials and meta-analyses have established that: Single-fraction radiotherapy (8 Gy) provides pain relief in 60%–70% of patients with bone metastases (Hovenier et al., 2025). Multi-fraction regimens (20–30 Gy in 5–10 fractions) achieve similar overall response rates with potentially more durable control (Imano et al., 2023). Complete pain relief occurs in 20%–35% of patients. Response typically occurs within 2–4 weeks, with maximal effect by 8–12 weeks (Hovenier et al., 2025). Iodine-125 (I-125) particle stereotactic ablative brachytherapy (SABT) demonstrates favorable local control outcomes. Studies report high objective response rates (ORR) and disease control rates (DCR), with concurrent significant improvements in pain relief and functional status (Chen Y. et al., 2023). While cement provides more rapid pain relief (within days), radiotherapy’s onset of action is still clinically acceptable for most patients without imminent structural failure.

#### 7.1.3 The risk-benefit calculus: a personalized approach

The choice between cement and radiation-based approaches should consider:

Favoring Cement: Immediate structural failure or impending fracture with high risk. Severe intractable pain requiring urgent relief. Prior radiation to tolerance of the area. Radioresistant tumor histology. Very short life expectancy where immediate palliation is paramount. Patient preference after informed discussion. Favoring Radiotherapy: Structurally intact bone without imminent fracture risk. Pain manageable with analgesics for weeks needed for radiation response. Radiosensitive tumor types (breast, prostate, myeloma, lymphoma). Oligometastatic disease with curative intent. Long life expectancy (>1 year) where long-term outcomes matter. Younger patients where preserving normal tissue architecture is valuable. Potential for bone healing and structural recovery. Concerns about mechanical effects on tumor biology.

### 7.2 Limitations of radiotherapy

Nevertheless, radiotherapy also has important limitations. Its analgesic effect is often delayed compared with the immediate symptomatic benefit of cement augmentation, and it does not provide instant mechanical stabilization in patients with impending or established pathological fracture. In addition, treatment response varies according to tumor histology, lesion location, and prior irradiation, and radiation-related toxicities or dose constraints may limit its applicability in some cases. Furthermore, structural recovery after radiotherapy is gradual and may be incomplete, such that some patients remain at risk of persistent mechanical pain or subsequent skeletal complications during follow-up.

In addition to external beam radiotherapy and stereotactic radiotherapy, other non-cement treatment options for metastatic bone disease should also be acknowledged. Contemporary reviews have highlighted the potential role of radiopharmaceuticals, particularly in patients with multifocal painful osteoblastic or mixed bone metastases, as well as selected image-guided palliative interventions such as embolization, thermal ablation, and high-intensity focused ultrasound in carefully chosen clinical scenarios. However, these approaches differ in indication, mechanism, and evidence base, and a comprehensive comparison is beyond the scope of the present hypothesis paper. Our central point is that, for patients without immediate structural instability, local treatment selection should not be framed solely around cement augmentation, and non-mechanically disruptive alternatives deserve explicit consideration.

## 8 Conclusion

This hypothesis is not intended to challenge the established and indispensable role of bone cement in patients with acute mechanical instability, established pathological fracture, or imminent structural collapse, where immediate stabilization is essential. Rather, it questions whether bone cement should be routinely regarded as the default local treatment for all symptomatic osteolytic metastases, particularly in patients with favorable prognosis, limited metastatic burden, and no urgent need for mechanical reinforcement. For oligometastatic patients with limited tumor burden, favorable prognosis, and radiosensitivity, the goal of local therapy has subtly evolved beyond mere pain relief and should encompass the pursuit of long-term local disease control and the maintenance of the natural biomechanical homeostasis of bone. Within this specific population, the theoretical advantages of radiotherapy—particularly stereotactic body radiotherapy (SBRT)—which effectively inactivates tumors while enabling bone self-repair, merit more prominent evaluation. Currently, clinical practice should adopt a balanced and prudent strategy based on the principle of evidence hierarchy. Until conclusive evidence from prospective studies is obtained, we recommend incorporating this mechanobiological perspective into informed consent and individualized decision-making during multidisciplinary team (MDT) discussions and shared decision-making between clinicians and patients.

For clinically stable patients without urgent indications for mechanical stabilization, a radiotherapy-based initial management strategy may be a reasonable subject for future prospective evaluation, rather than an evidence-based standard of care at present. Looking ahead, verifying or refuting this hypothesis will require concerted, interdisciplinary collaboration. This includes developing advanced preclinical models that can simulate the microenvironment of bone cement stiffness, conducting prospective clinical cohort studies comparing the long-term local control rates between bone cement and radiotherapy, and exploring correlations between mechanical parameters and tumor biological markers through finite element analysis and molecular imaging. The main value of the present work lies in hypothesis generation rather than practice change. We do not propose that current treatment algorithms should be altered on the basis of the available evidence, nor do we suggest that radiotherapy should replace bone cement in routine practice. Instead, we argue that the long-term mechanobiological consequences of cement-induced stiffness changes represent an underexplored question that may be particularly relevant in selected clinically stable patients with longer expected survival. This question warrants testing in bone-specific experimental systems and prospective clinical studies before any treatment prioritization can be justified.

## Data Availability

The original contributions presented in the study are included in the article/supplementary material, further inquiries can be directed to the corresponding authors.
